# Motivation and personality factors of Generation Z high school students aspiring to study human medicine

**DOI:** 10.1186/s12909-021-03099-4

**Published:** 2022-01-11

**Authors:** Barbara M Holzer, Oriane Ramuz, Christoph E Minder, Lukas Zimmerli

**Affiliations:** 1 Department of Internal Medicine, University Hospital Zurich, Zurich, Switzerland; 2 Department of Internal Medicine Cantonal Hospital Olten, Solothurner Spitäler AG, Olten, Switzerland

**Keywords:** High school students, Generation Z, Human medicine, Motivation to study medicine, Career motivation, Personality traits, Life goals

## Abstract

**Background:**

A new generation of medical students, Generation Z (Gen Z), is becoming the predominant population in medical schools and will join the workforce in a few years’ time. Medicine has undergone serious changes in high-income countries recently. Therefore, it is unclear how attractive the medical profession still is for high school students of Gen Z. The aim of this study was to investigate what motivation leads Gen Z students in their choice to study human medicine, and how they see their professional future. Our study was guided by motivation theory and the influence of personality traits and other personal factors on students’ choice of university major.

**Methods:**

In a cross-sectional online survey, we included third- and fourth-year high school students in Northern Switzerland. We examined the importance of criteria when choosing a university major: personality traits, career motivation, life goals, and other considerations influencing the choice of human medicine versus other fields of study. Results Of 1790 high school students, 456 (25.5%) participated in the survey (72.6% women, mean age 18.4 years); 32.7% of the respondents aspired to major in medicine at university. For all respondents, the foremost criterion for selecting a field of study was ‘interest in the field,’ followed by ‘income’ and ‘job security.’ High school students aiming to study human medicine attached high importance to ‘meaningful work’ as a criterion; supported by 36.2% of those students answering that helping and healing people was a core motivation to them. They also scored high on altruism (*p* < 0.001 against all groups compared) and intrinsic motivation (*p* < 0.001) and were highly performance- (*p* < 0.001) and career-minded (*p* < 0.001). In contrast, all the other groups except the law/economics group had higher scores on extraprofessional concerns.

**Conclusions:**

Swiss Gen Z students aspiring to study human medicine show high intrinsic motivation, altruism, and willingness to perform, sharing many values with previous generations. Adequate work-life balance and job security are important issues for Gen Z. Regarding the current working conditions, the ongoing shortage of physicians, and recent findings on physicians’ well-being, the potential for improvement and optimization is high.

**Supplementary Information:**

The online version contains supplementary material available at 10.1186/s12909-021-03099-4.

## Background

Like many other high-income countries, Switzerland faces a relative shortage of physicians, moreover 15% of primary care physicians are of retirement age. The next generation of physicians is urgently needed [1]. Many residents of the current generation (Generation Y, or Millennials, born 1980 to 1995) are not satisfied with their work-life balance, and they seem to be a vulnerable population regarding mental well-being, burnout, and quitting patient care, as found by various Swiss [2–4] and international studies [5–7]. A new generation of students, Generation Z (Gen Z, born 1995 to 2010), is becoming the predominant population at university and will join the workforce in a few years’ time [8]. They grew up during a time of socioeconomic uncertainty and complexity and were educated in settings that promoted inclusivity and diversity [9]. Gen Z are said to be activists, to be highly motivated regarding their education and profession, and to seek meaningful activity rather than social prestige [10, 11]. As any choice of study major, the choice to study medicine and the underlying motivation depend upon various factors, such as interest in the field, job opportunities, a desire to serve others, and many more [12].International research on the motivation of medical students and physicians working in direct patient care is diverse, due to different perspectives and outcomes [13–17].

Self-determination theory (SDT) has been frequently used to examine factors influencing the motivation to study and practice human medicine in high school students, medical students, and active physicians. SDT of motivation distinguishes between intrinsic and extrinsic motivation. According to SDT, intrinsic motivation contains motivation goals such as interest in the field, interest in human relationships, helping people, looking for meaningfulness. Intrinsic motivation depends on the fulfilment of three basic psychological needs, the need for autonomy, competence, and relatedness [18]. Extrinsic motivators, such as having a career [19, 20], financial security, or prestige and status [20–23], have also been reported as important for choosing medical studies, with differences by gender and socioeconomic factors [24].

In Switzerland, career motivation, life goals, and work-life balance of medical students and physicians and their career paths have been investigated and followed for many years [23, 25–28]. Buddeberg-Fischer’s findings indicated that gender and personality traits, as well as strong intrinsic and extrinsic career motivation, contribute to academic achievement and career planning in Swiss medical students [23]. In addition, Swiss high school students showed a certain degree of stability in sense of coherence (SOC) [25]. The concept of the SOC was introduced by Antonovsky in his model of salutogenesis [29]. The SOC is a global orientation and reflects the extent to which individuals perceive their life as comprehensible, manageable and meaningful. More precisely, the SOC scale measures the extent to which an individual is likely to construe a stressor as comprehensible and worth overcoming, and the individual's appraisal that he or she will manage to overcome such stressors [25, 29, 30]. That means, a strong sense of coherence helps one mobilize resources to cope with stressors and manage tension successfully [31]. All of the above-named factors are favorable for surmounting the high barriers to medical school entrance and the obstacles during medical education. After graduating from high school, students can choose any field of study at Swiss universities without an entrance exam except medicine. In Switzerland, medicine is the only course of study requiring an aptitude test, which is “designed to measure the potential to follow and succeed in medical studies” [32] p. 2

Facing the current conditions in health care provision/health services with increasing economization, cost pressure, and stress and burnout, and physicians leaving direct patient care as mentioned above, it is unclear what motivates members of Gen Z to choose a medical profession.

In this study, we investigated how current Gen Z high school students view their future field of study, i.e., what considerations guide them in their choice to study human medicine, and how they see their professional future. We also investigated the influence of personality traits and other personal factors on their choice of major. Our hypotheses were guided by motivation research in medical education [13], in particular by SDT [33] and research in Switzerland by Buddeberg-Fischer and colleagues [2, 26, 27]. Regarding career orientation, we hypothesized (1) that future medical students tend to have a more pronounced intrinsic motivation than students aspiring to study other fields. In addition, we hypothesized (2) that performance- and career-orientation in terms of seeking competence and autonomy, as well as leadership are important issues for high school students aspiring to study medicine. Given the fact of the many obstacles that medical students have to overcome passing an aptitude test and the long duration of medical education and post-education, we further hypothesized (3) that students aspiring to study medicine tend to have a high SOC.

## Methods

### Population and survey

To initiate this cross-sectional anonymous online survey an information letter was sent to the head teachers at all eight high schools in the Cantons of Aargau and Solothurn, Switzerland in March 2019. In a second step, an e-mail explaining the aim and purpose of the study in detail was sent to the same head teachers in April 2019. The head teachers were asked to forward the e-mail, with a link to the online survey, to their third- and fourth-year high school students, i.e., the students graduating in that or the following year. Three head teachers declined participation in the survey. Thus, a potential of 1790 high school students were addressed between April 29 and May 21, 2019.

### Questionnaire and data collection

Besides gender, age, grade, and school affiliation, we asked survey respondents whether they intended to study human medicine after having obtained their *Matura* (Swiss university entrance qualification). Students who did not consider majoring in human medicine were asked what other field they aimed to study, or if they were still undecided or not intending to pursue university studies at all.

In the next section of the questionnaire, the students had to rate the importance of 10 criteria in choosing their university major or profession. Prospective medical students were asked why they were interested in studying human medicine (open-ended question: “What is fascinating to me in human medicine is…”). In order to obtain results comparable with the findings of Buddeberg-Fischer [26] and Cribari [2], we applied the same measurement instruments for personality traits and other personal factors as those used by Buddeberg-Fischer and colleagues [23, 26–28]. Thus, the next section contained items on personality and career-related factors from the Career Motivation Questionnaire (CMQ, 24 items), the Life Goals questionnaire (GOALS, 24 items), a questionnaire on perception of the future (five items), and the Sense of Coherence scale (SOC, 13 items).

In addition, for students aspiring to study human medicine we were interested in their personal view of their future professional standing in 15 years, i.e., whether they saw themselves working in a hospital, as a specialist in private practice, as a general practitioner, in medical research, or in management or consultancy positions. We chose 15 years, as in Switzerland length of medical studies is 6 years and postgraduate medical training is another 5 to 8 years, depending on the specialty aimed for. Further, we were interested in the students’ opinion on the official weekly work time for residents, which in Switzerland is currently 50 h.

Questionnaires were eligible to be included in the analyses if the respondents had filled out at least all variables of participants’ characteristics and the rating of criteria in choosing their university major or profession. The study was conducted in compliance with the STROBE guidelines of observational studies [34]. Participation in this online survey was completely anonymous and entirely on a voluntary basis. The research team had no access to the email addresses of the respondents. Data were collected using SurveyMonkey online software (www.surveymonkey.de) and transferred to a spreadsheet in Microsoft Excel Version 2016 (Redmond, WA, USA) for data management.

### Statistical methods

For two-group comparisons, tabulations and chi-square tests as well as t-tests were used. Analysis of variance (ANOVA) was used for simultaneously comparing graduated variables over all five fields of study. Personality related factors, such as SOC and CMQ, were defined as described in Buddeberg-Fischer et al. [26]. T-tests, confidence intervals, and Cohen’s effect size were used to assess the relative importance of the various factors influencing the choice of medical studies as opposed to other university majors. Chi2-test was used to examine gender differences.

We used content analysis, a qualitatively oriented category-guided text analysis, for the respondents’ electronic answers to the free-text question [27, 37]. The two main steps of the content analysis were first, the development of a categorization frame with a code manual and determining operational definitions for each content category, and second, coding the text according to the categorization frame to the content categories.

For data analyses we used Stata Statistical Software: Release 16 (StataCorp, College Station, TX, USA) and IBM SPSS Statistics Version 25 (IBM Corp, Armonk, NY, USA). The significance level was set at 0.05, two-sided.

## Results

A total of 643 high school students participated in the survey (response rate 35.9%). We excluded 187 questionnaires because they were incomplete or not interpretable. Questionnaires completed by 456 (25.5%) respondents were included in the analyses.

Study participants were on average 18.4 years old; 72.6% were young women (Table 1). When asked what they planned to major in at university, 32.7% of the respondents aimed to major in human medicine, 21.9% in fields within the faculty of arts and sciences, 21.5% in natural sciences or technical fields, 12.5% in law or economics; 11.4% of the respondents were undecided or intended to pursue other professional education tracks.

**Table 1:** Study population of this online survey

Participants *N* = 456		
**Age in years, mean (SD)**		18.38 (1.06)
**Gender**	N	%
Male	125	27.41
Female	331	72.59
**School affiliation**		
Aarau (old)	108	23.68
Baden	99	21.71
Wettingen	126	27.63
Zofingen	59	12.94
Other venues (Aarau new, Wohlen, Olten, Solothurn)	64	14.04
**Planned major at university**		
Human medicine	149	32.68
Faculty of arts & social sciences	100	21.93
Natural sciences, technical fields	98	21.49
Law/economics	57	12.50
Other/undecided	52	11.40

In their ratings of 10 criteria for choosing a university major, ‘interest in the field’ was for all students the most important criterion followed by ‘income’ and ‘job security’ in second or third position, depending on the field of study in which they planned to major (Table 2). As an exception, students aspiring to study human medicine rated ‘meaningful work’ as just as important as ‘job security’ and as more important than ‘income.’ Comparing the group of students aiming to major in human medicine with four groups choosing other fields of study, the most obvious differences were found with students aiming to major in law or economics. Whereas ‘meaningful work’ as well as ‘teamwork’ and ‘opportunity to work independently’ were rated highly by those aiming to study human medicine, ‘income’ and ‘career opportunities’ were rated highly by those aiming to study law/economics. Comparing students choosing medicine with those aiming for natural sciences/technical fields, the only significant difference was in ‘length of studies,’ which was a criterion that was more important to students choosing to major in natural sciences/technical fields.

**Table 2:** Respondents’ average rating of the importance of 10 criteria for choosing a university major, grouped by field of study aimed for. All tests were comparisons with human medicine

Criteria for choosing field of study	Human medicine	Faculty of arts & social sciences	Natural sciences, technical fields	Law/economics	Other/undecided	*P*-value Anova
	***N* = 149**	*N* = 100	*N* = 98	*N* = 57	*N* = 52	
Interest in the field	8.32	8.95	7.98	8.49	8.44	0.250
Job security	6.55	6.38	6.37	6.82	6.77	0.603
Income	6.01	6.27	6.48	7.53	6.35	0.002
Meaningful work	6.55	5.93	4.87	4.51	5.29	< 0.001
Career opportunities	5.22	4.85	4.86	6.32	5.25	0.004
Opportunity to work independently	5.11	5.25	4.87	4.23	4.65	0.086
Teamwork	5.11	5.17	5.23	4.25	5.04	0.133
Working hours	4.36	4.63	4.87	4.26	5.04	0.220
Social prestige	4.33	3.67	4.35	5.14	3.44	0.006
Length of studies	3.46	3.90	4.18	3.46	4.73	0.013

Respondents’ rating of the importance of 10 criteria for choosing a university major, grouped by field of study aimed for. Response options from "1 = not important at all" to "10 = very much important"

ANOVA indicated that there were highly significant group differences for ‘meaningful work,’ ‘income,’ ‘career opportunities,’ ‘social prestige’ and barely significant differences for ‘length of studies.’ More than for all other groups ‘meaningful work’ is a criterion of great importance to those students aspiring to study medicine. Analyzing personality factors of the respondents, we first tested human medicine against the four other groups defined (Table 3). We found indications for some considerable differences between human medicine and the other groups.

**Table 3:** Personality traits (personality and career-related factors, means) of high school students grouped according to their choice of university major. Part 1: Means and ANOVA test

Part 1: Means and Anova	Human medicine	Faculty of arts & social sciences	Natural sciences, technical fields	Law/economics	Other/undecided	*P*-value Anova
	*N* = 149	*N* = 100	*N* = 98	N = 57	N = 52	
Career Motivation*						
CMQ Intrinsic motivation	5.87	5.58	5.57	5.55	5.33	< 0.001
CMQ Extrinsic motivation	4.34	4.13	4.06	4.73	4.15	< 0.001
CMQ Extraprofessional concerns	3.68	4.19	4.00	3.81	4.01	< 0.001
Importance of Life Goals**						
Intimacy	4.40	4.42	4.44	4.31	4.33	0.772
Affiliation	3.59	3.52	3.31	3.42	3.46	0.176
Altruism	4.32	3.94	3.89	3.77	3.94	< 0.001
Power	2.88	2.92	2.67	3.38	2.56	< 0.001
Achievement	4.30	4.08	4.09	4.08	3.95	0.003
Variation	4.29	4.17	4.01	4.28	4.20	0.097
Perceptions of the future***						
Leading position as a main goal	3.44	2.89	2.97	3.54	2.73	< 0.001
Challenges in work but also time for other things	4.58	4.70	4.63	4.41	4.58	0.317
Part-time work to have more private time	3.82	4.26	3.89	3.59	3.82	0.012
May leave work to do something else	1.80	2.37	2.16	2.07	2.47	0.002
Reduction of work to temporarily have more family time	3.04	3.50	3.37	2.98	3.62	0.003
**Sense of Coherence (SOC)**	4.58	4.38	4.41	4.47	4.41	0.305

Career motivation*: Response options from 1 = *never* to 7 = *very often*, Intrinsic motivation (i.e. interest in professional activities, autonomy, relatedness), Extrinsic motivation (i.e., striving for promotion, income, social prestige), Extraprofessional concerns (i.e., starting a family, convenient working hours)

**Importance of life goals** (six major life domains)**: Response options from 1 = *not important* to 5 = *very important*, Intimacy (close relationship based on mutual trust and affection), Affiliation (spending time with other people, common activities), Altruism (acting for the welfare of others), Power (asserting oneself, seeking social status), Achievement (improving on oneself, meeting standards), Variation (seeking new experiences and excitement)

Perceptions of the future***: Response options from 1 = *not at all* to 5 = *completely*

Looking closer at career motivation, students aspiring to study human medicine had a significantly higher intrinsic motivation than the average in all other groups (Table 4, Cohen’s effect estimate: 0.526, *p* < 0.001, t-test), and thus these findings supported our hypothesis 1.

**Table 4:** Personality traits (personality and career-related factors, means) of high school students grouped according to their choice of university major. Part 2: Comparisons of human medicine against all others (t-test, difference and Cohen's effect size)

Part 2: Comparisons of human medicine against all others t-test,difference und Cohen's effect size)	Human medicine	All other disciplines together (except human medicine)	Difference	CI low	CI up	Cohen's effect size	*P*-value t-test
	*N* = 149	*N* = 307					
Career Motivation*							
CMQ Intrinsic motivation	5.87	5.53	0.338	0.207	0.470	0.526	< 0.001
CMQ Extrinsic motivation	4.34	4.23	0.111	-0.058	0.280	0.138	0.197
CMQ Extraprofessional concerns	3.68	4.03	-0.346	-0.517	-0.175	-0.432	< 0.001
Importance of Life Goals**							
Intimacy	4.40	4.39	0.152	-0.126	0.157	0.024	0.832
Affiliation	3.59	3.42	0.165	-0.009	0.339	0.211	0.064
Altruism	4.32	3.89	0.431	0.278	0.583	0.608	< 0.001
Power	2.88	2.86	0.024	-0.168	0.216	0.028	0.808
Achievement	4.30	4.06	0.245	0.118	0.373	0.425	< 0.001
Variation	4.29	4.14	0.148	-0.011	0.306	0.208	0.068
Perceptions of the future***							
Leading position as a main goal	3.44	3.01	0.429	0.197	0.661	0.398	< 0.001
Challenges in work but also time for other things	4.58	4.60	-0.021	-0.179	0.137	-0.029	0.795
Part-time work to have more private time	3.82	3.94	-0.129	-0.374	0.116	-0.115	0.151
May leave work to do something else	1.80	2.27	-0.467	-0.715	-0.218	-0.405	< 0.001
Reduction of work to temporarily have more family time	3.04	3.39	-0.344	-0.593	-0.094	-0.299	0.007
**Sense of Coherence (SOC)**	4.58	4.41	2.026	0.075	3.976	0.217	0.042

According to our results, altruism was a very important motivational factor for young people to study medicine and thus reached a significantly and considerably higher score than the average of all other student groups (Cohen’s effect estimate: 0.608, *p* < 0.001, t-test).

**Fig. 1:**
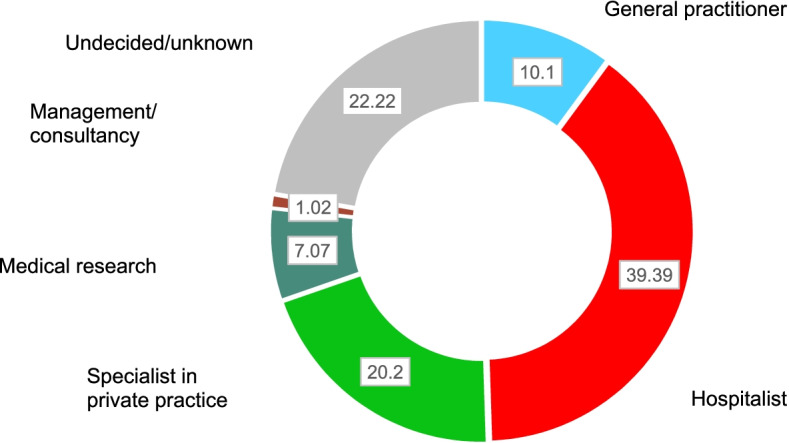
Professional standing in 15 years in the personal view of interviewed high school students aspiring to study human medicine (in %, *N* = 83)

Nevertheless, students aiming to study medicine had a high performance orientation in terms of professional competence and interest in continuing education and quality improvement. Thus, ‘achievement’ (improving oneself, meeting standards) was an important life goal and was rated significantly higher for this group than for all other groups compared (Cohen’s effect estimate: 0.425, *p* < 0.001, t-test). Similar to this, attaining a leading position as a main goal in the future had significant importance for medical aspirants, with a higher score than for the average of all other goals (Cohen’s effect estimate: 0.398, *p* < 0.001, t-test). In contrast, aspiring medical students had less interest in extraprofessional concerns than students choosing all other fields of study together (Table 4). According to our hypothesis 2, these results highlight their high performance- and career orientation. Scores on the SOC showed no significant differences across fields of study (Anova, *p* = 0.305), even though the human medicine group had a marginally higher SOC than all other fields of study combined (Cohen’s effect: 0.217, *p* = 0.042, t-test, Table 4). Nevertheless, these results did not support our hypothesis 3. When asked why they were interested in studying human medicine (Table 5), 36.2% of the respondents aiming to study medicine stated that it was the prospect of helping and healing people, 32.5% were particularly interested in the field of human medicine and its complexity, 15.7% aimed to gain a deep understanding of the human body and its functioning, 9.6% named continuous education and personal development (improving oneself) as the reason for their interest in studying medicine, and 6.0% gave other explanations for their particular interest, such as interest in the ability to adapt and modify the human body to a certain extent. Regarding the respondents’ personal view of their long-term professional standing (Fig. 1), 39.4% of the students aspiring to study human medicine stated that they wanted to work at a hospital, 20.2% as a specialist in private practice, 10.1% as a general practitioner, 7.1% in medical research, and 1.0% in a management or consultancy position; 22.2% remained undecided or unclear. Of a total of 149 medical aspirants participating in the survey, 83 (56%) did answer to the open questions listed in Table 5 and Fig. 1.

**Table 5:** Free text responses of high school students aspiring to study human medicine,: “What is fascinating to me in human medicine is …”. Categorization of the answers (initial entry only). *N* = 83

**Motivation to study human medicine**	*N*	%
Qualification to help and to heal people	30	36.2
Continuous education, lifelong learning	8	9.6
Complexity of the discipline human medicine	27	32.5
Gaining a deep understanding of the human body	13	15.7
Other reasons	5	6.0

Prospective medical students were asked about the weekly work time of 50 h for residents in Switzerland; 65.1% rated this workload as being part of normal professional life, 23.5% rated this weekly workload as too heavy and proposed an average a work time of 42.5 h; 11.4% of the students had no opinion on this issue. We found gender differences in this study population. Across all types of university majors, young men rated ‘income’ (*p* = 0.007), ‘social prestige’ (*p* = 0.024), and ‘career opportunities’ (*p* = 0.033) as more important than young women did, whereas women rated the criterion ‘length of studies’ (*p* = 0.010) as more important in choosing a university major than men did. There were no gender differences in any of the other criteria assessed. We also found some gender differences in personality factors within the total study population: Young women had higher scores on altruism (*p* < 0.001). In contrast, young men scored higher on extrinsic motivation (*p* = 0.033). There was a particularly obvious difference in perceptions of the future. Women were more often open to challenges outside work (*p* < 0.001) as well as to part-time work (*p* = 0.002), or to reducing work temporarily to have more family time (*p* = 0.019). In contrast, men aimed more often for a work break to do something else (*p* = 0.014). A gender analysis among students aspiring to study medicine was not performed, due to the relatively small proportion of men in this group.

## Discussion

### Main results

Our cross-sectional anonymous online survey in Northern Switzerland reveals that Gen Z high school students aiming to study human medicine at university show a high intrinsic career motivation, attach high importance to ‘meaningful work’ as a criterion for choosing human medicine for their future profession, and in particular score high on altruism. Moreover, about a third of the students answering declare that helping and healing other people is their main motivation for choosing this major. Nevertheless, these Gen Z students are also highly performance- and career-minded.

Students choosing other university majors, with the exception of the law/economics group, tended on average to attach more importance to extraprofessional concerns and a good work-life balance. The prospective students of law/economics also showed a strong career orientation but with a focus on an extrinsic motivation, such as ‘income’ and ‘social prestige.’ Gender analyses reveal that young women tend to aim more for a good work-life balance, in particular regarding family life. Taken together, our findings indicate strong personal commitment on the part of Gen Z students aiming to study human medicine at university.

### Context

The results of this survey of students aiming to study human medicine at university fit well with the findings of two other Swiss studies on Generation Y medical residents [2] and Generation X doctors [26]. All three studies, which used the same instruments to measure personality factors, showed high agreement between the three groups representing successive generations. Still, members of Gen Z present higher scores on altruism and seeking new experiences (‘variations’), and lower scores on extraprofessional concerns than Generation Y medical residents and Generation X doctors. These differences in life goals and career motivation are probably due to the young age of the Gen Z population and their lack of specific professional experience in contrast to residents 10 years older, whose enthusiasm might have been dampened to some extent by the experience of working in acute care hospitals.

Like the previous generations of physicians, Gen Z future medical students showed high levels of intrinsic motivation. In comparison to other students of the same age, future medical students showed by far the highest intrinsic motivation of all groups included. High levels of altruism and aspects of altruistic motives such as ‘meaningful work’ as the most important factor in choosing human medicine and the personal statement of the core motivation to ‘help and to heal people’ support their high intrinsic motivation. The intention to help people has been found in the literature to be a very strong motivation to study human medicine [13, 38]. Ratanawongsa et al. stated that for students entering medical school, the desire to help others or for intellectual challenge through medicine may overweigh the potential trade-offs in immediate lifestyle or salary [38]. According to SDT, quality of motivation is considered to be more important than quantity [18, 39]. Intrinsic motivation makes a person pursue an activity out of personal interest or for enjoyment and is the most self-determined form of motivation [13]. A study in the United States showed that intrinsic motivators were associated with physician well-being and commitment to clinical practice, whereas extrinsic factors such as income and work-related characteristics were not associated with meaning or commitment [40]. Tak et al. conclude that career commitment might be improved by cultivating a sense of vocational identity while promoting a work environment in which physicians experience their work as being personally rewarding [40]. Compared to the two previous generations, Gen Z high school students aspiring to study medicine have the highest extrinsic motivation scores on career motivation but lower scores on extraprofessional concern [2, 26]. Nevertheless, almost one quarter of these Gen Z students rate the weekly workload of 50 h for residents as too heavy and propose an average work time of 42.5 h, which in Switzerland is close to the average work time for other professions (40–42 h). But the reality is different: A time and motion study on internal medicine residents in a Swiss hospital reported day shifts lasting 1.6 h longer than scheduled [41]. Job security was rated just as important as meaningful work in the criteria for choosing to study medicine. An annual survey in 2019 conducted by Universum, an international employer branding firm, revealed the highest percentage of students seeking job security of the last 10 years [42]. It is unclear whether this sentiment among students is a consequence of their perceptions of the employment outlook or a sign of greater conservatism among Gen Z compared to Generation Y [42]. For Gen Z, dedication to a cause and leadership seem to be more important issues than for the previous generation [43]. In our survey, attaining a leading position was also an important issue for future medical students, with a higher score than for the average of all other students. According to Stillman and Stillman, 74% of Gen Z are motivated by prospects of career advancement and receiving credit for doing a good job [44]. SOC is a widely recognized measure of an individuals’ resistance to stress. It develops in childhood and adolescence and then keeps quite stable during the life course [29]. In a study concerning personal predictors to manage stress by university students the SOC showed to be a very important trait for stress coping strategies [45]. Although students aspiring to study medicine had the highest mean SOC score of all groups examined, we could not find a significant difference between the five groups of high school students. Other surveys presented a similar mean SOC score in high school students of the same age in the Canton of Zurich [25] and in an adolescent group in a German survey [46]. However, a recent report on SOC in first-year medical students found human medicine students with significantly lower means of SOC in comparison to students in dentistry and molecular medicine [47].

Provided policy makers and health care directors succeed in preserving the initially high intrinsic motivation by improving physicians’ working conditions, such efforts would probably help to prevent physicians from leaving the field of medicine [5, 43]. Hospitals are particularly challenged, as almost all Swiss physicians complete their residency in hospitals and 46% work in hospitals [48]. In our survey, more than a third of future Gen Z physicians see their professional future in a hospital. Most physicians make their career choices early on during residency [48], and during this period a considerable proportion of them leave the field of medicine [3]. To keep the medical profession attractive for the future, a first step might be to obtain more insight into the reasons for highly stressful working conditions, so as to gain a better understanding of challenges to clinician well-being. Organizations can then aim to implement evidence-based solutions to avoid and relieve stress at work and to monitor the solutions’ effectiveness [49]. A recent qualitative study from the Netherlands has shown that medical specialists' motivation was negatively influenced by mainly tasks and organizational processes that distract from patient care or that compromise the quality of care [50]. According to the authors, a work environment including sufficient time for patients and teaching optimizes motivation in daily work [50]. In our survey respondents, adequate work-life balance, reducing work temporarily to have more family time (women), and having a work break (men) are also important issues and reflect the current trend of the young generation. This is in line with a recent Swiss survey: Berchtold et al. found that family time, vacation, and career breaks for private reasons were the main reasons for female physicians to take longer postgraduate training [51]. For male physicians the main reasons were additional training and scientific work. Innovative career models and flexible work conditions have to be developed that allow for an adequate work-life balance for physicians of both genders. Furthermore, the organization’s altruistic mission statement has to align with the institution’s actions and policies [52]. A primary focus for organizations should be to optimize the practice environment and create a healthy organization culture, as organizational-level efforts can have a profound effect on physician well-being [53].

All these efforts help to maintain the physical and psychological health and the high level of motivation of Gen Z prospective physicians and other health care professionals during their professional life. The resulting cultural change towards more communication, which matters to the health care workers, as well as less hierarchy among professions in health care seems to accommodate Gen Z, which is said to be a generation of activists and dialoguers.

### Limitation and strengths

Our study has several limitations. First, the response rate was relatively low, indicating the possibility of response bias. Nevertheless, a response rate of 25% is not unusually low for this kind of survey [2, 54]. Second, the proportion of high school students intending to study medicine was very high compared to other fields of study. Our survey may have addressed and activated more students with an affinity to human medicine than other students. Third, three quarters of the respondents were young women, an overrepresentation that is often found in similar surveys [2, 55]. We have taken account of this by looking at certain gender differences. Another limitation is the young age of the respondents, which might have influenced the validity/explanatory power of the personality factors under study.

As for the strengths of our study, to our knowledge this is the first survey analyzing personal and individual factors likely influencing the choice of human medicine as a university major in Gen Z. The survey was conducted completely anonymously, and non-participation led to no disadvantages for the students. Moreover, bias due to social desirability of the respondents’ answers should have been brought down to a minimum. In addition, the comparison of the results of three studies using the same personality tests revealed good agreement.

## Conclusions

Gen Z students aspiring to study human medicine at university show high intrinsic motivation and altruism and high willingness to perform, sharing many values with previous generations. Nevertheless, they are a potentially vulnerable population. With the current working conditions in medicine, the ongoing shortage of physicians, and recent findings on physicians’ well-being in Switzerland, the potential for improvement and optimization, in particular for young residents, is high. In the light of these results, workplace conditions have to change in a direction that supports values and preferences of young students in their high intrinsic motivation, according to SDT. Motivation guidance should not only focus on the professional career but also consider personal life goals and enable a good work-life balance, taking into account that for both women and men, motivating factors vary during different career stages.

## Supplementary Information

**Additional file 1.**

## Data Availability

The datasets used and/or analyzed during the current study are available from the corresponding author on reasonable request.

## Abbreviations

CMQ: Career Motivation Questionnaire
FADP: Federal law on data protection
Gen Z: Generation Z
GOALS: Life Goals questionnaire
HRA: Human Research Act
SOC: Sense of Coherence
SDT: Self-determination theory
