# Supplementary material for: Motivation and personality factors of Generation Z high school students aspiring to study human medicine
Source: BMC Med Educ. 2022 Jan 11;22:31. doi: 10.1186/s12909-021-03099-4 (PMC8753872; doi:10.1186/s12909-021-03099-4)
Supplement: Supplementary file 1 — Additional file 1. [file 12909_2021_3099_MOESM1_ESM.docx]

**Supplementary material**

**English translation of the online survey used for the study: “How about going into medicine? Motivation and personality factors of Generation Z high school students aspiring to study human medicine: a cross-sectional online survey in Northern Switzerland”**

Barbara M. Holzer^a^, Oriane Ramuz^b^, Christoph E. Minder^b^, Lukas Zimmerli^b^

^a^ Department of Internal Medicine, University Hospital Zurich, Switzerland

^b^ Department of Internal Medicine, Solothurner Spitäler AG, Kantonsspital Olten, Switzerland

**Corresponding author**

Lukas Zimmerli, MD

Department of Internal Medicine

Solothurner Spitäler AG

Kantonsspital Olten

Baslerstrasse 150

CH- 4600 Olten

lukas.zimmerli@spital.so.ch

**Demographic data**

Gender:

□ Female

□ Male

Age (in years): ___

School affiliation:

□ Alte Kantonsschule (old cantonal high school, Aarau)
□ Neue Kantonsschule (new cantonal high school, Aarau)
□ Baden
□ Wettingen
□ Wohlen
□ Zofingen
□ Olten
□ Solothurn

How likely is it that you will go on to study human medicine after graduating from high school?

□ Very likely

□ Rather likely

□ Rather unlikely

□ Not likely at all

For students who do not consider majoring in human medicine: What other field of study are you aiming for?

□ Faculty of arts and social sciences

□ Natural sciences, technical fields

□ Law/economics

□ Other/undecided

**Rating of 10 criteria for choosing a university major**

Please rate the importance of each of the following 10 criteria for choosing a university major on a scale from 1 = most important to 10 = least important

Length of studies ___

Social prestige ___

Working hours ___

Teamwork ___

Opportunity to work independently ___

Career opportunities ___

Meaningful work ___

Salary ___

Job security ___

Interest in the field ___

**Questions about different aspects of life (SOC-13)**

| 1. Do you have the feeling that you don't really care about what goes on around you? | | | | | | | | |
| --- | --- | --- | --- | --- | --- | --- | --- | --- |
| never | □ | □ | □ | □ | □ | □ | □ | very often |
| 1. Has it happened in the past that you were surprised by the behavior of people whom you thought you knew well? | | | | | | | | |
| never happened | □ | □ | □ | □ | □ | □ | □ | always happened |
| 1. Has it happened that people whom you counted on disappointed you? | | | | | | | | |
| never happened | □ | □ | □ | □ | □ | □ | □ | always happened |
| 1. Until now your life has had: | | | | | | | | |
| no clear goals | □ | □ | □ | □ | □ | □ | □ | very clear goals and purpose |
| 1. Do have the feeling that you're being treated unfairly? | | | | | | | | |
| very often | □ | □ | □ | □ | □ | □ | □ | very seldom or never |
| 1. Do you have the feeling that you are in an unfamiliar situation and don't know what to do? | | | | | | | | |
| very often | □ | □ | □ | □ | □ | □ | □ | very seldom or never |
| 1. Doing the things you do every days is: | | | | | | | | |
| a source of deep pleasure and satisfaction | □ | □ | □ | □ | □ | □ | □ | a source of pain and boredom |
| 1. Do you have very mixed-up feelings and ideas? | | | | | | | | |
| very often | □ | □ | □ | □ | □ | □ | □ | very seldom or never |
| 1. Does it happen that you have feelings inside you would rather not feel? | | | | | | | | |
| very often | □ | □ | □ | □ | □ | □ | □ | very seldom or never |
| 1. Many people—even those with a strong character—sometimes feel like sad sacks (losers) in a certain situation. How often have you felt this way in the past? | | | | | | | | |
| very often | □ | □ | □ | □ | □ | □ | □ | very seldom or never |
| 1. When something has happened have you generally found that: | | | | | | | | |
| you overesti-mated or underesti-mated its importance | □ | □ | □ | □ | □ | □ | □ | you saw things in the right proportion |
| 1. How often do you have the feeling that there's little meaning in the things you do in your daily life? | | | | | | | | |
| very often | □ | □ | □ | □ | □ | □ | □ | very seldom or never |
| 1. How often do you have the feeling that you're not sure you can keep under control? | | | | | | | | |
| very often | □ | □ | □ | □ | □ | □ | □ | very seldom or never |

**Questions about job-related motivation**

*For each statement, please place a check mark in the appropriate box to indicate to what extent the statement applies to you personally.*

|  | very true of me |  |  |  | not true of me |
| --- | --- | --- | --- | --- | --- |
| I want to perform well professionally. | □ | □ | □ | □ | □ |
| I am also willing to take a risk on important decisions. | □ | □ | □ | □ | □ |
| I am convinced that I have the ability to achieve my desired professional goals. | □ | □ | □ | □ | □ |
| A secure job is more important to me than possible career advancement. | □ | □ | □ | □ | □ |
| When choosing my jobs, I make sure that they benefit my career. | □ | □ | □ | □ | □ |
| It is more important for me to be able to do what interests me than to advance professionally. | □ | □ | □ | □ | □ |
| I chose my field based on opportunities in the job market. | □ | □ | □ | □ | □ |
| I would travel for business any time. | □ | □ | □ | □ | □ |
| Pleasant working hours are important to me. | □ | □ | □ | □ | □ |
| In the future I would want in any case to keep my professional skills up to date. | □ | □ | □ | □ | □ |
| It would be terrible for me not to be able to achieve my desired professional goal. | □ | □ | □ | □ | □ |
| I look forward to familiarizing myself frequently with new professional tasks. | □ | □ | □ | □ | □ |
| In my profession I would like to work together with others. | □ | □ | □ | □ | □ |
| I would like to earn a lot of money in any case. | □ | □ | □ | □ | □ |
| I would put time into the job even at my spouse’s/partner's expense. | □ | □ | □ | □ | □ |
| It would be natural for me to work overtime. | □ | □ | □ | □ | □ |
| I would like to be able to bring my own ideas into the profession. | □ | □ | □ | □ | □ |
| I would accept difficulties in my marriage/partnership caused by my working life. | □ | □ | □ | □ | □ |
| It is important for me to achieve a socially respected professional position. | □ | □ | □ | □ | □ |
| It is no fun working under pressure. | □ | □ | □ | □ | □ |
| I want to take on responsibility in my profession. | □ | □ | □ | □ | □ |
| I will consistently work towards advancing my career. | □ | □ | □ | □ | □ |
| I would mind having to work weekends. | □ | □ | □ | □ | □ |
| It is not a high priority for me to advance professionally. | □ | □ | □ | □ | □ |

**Questions about your perception of your future in the next 10 years**

*For me, realizing this aim is…*

|  | not at all important |  |  |  | essential |
| --- | --- | --- | --- | --- | --- |
| I would like to work in a responsible management position or specialist position. For this, I am prepared to invest more than my required weekly work hours and to give up free time. | □ | □ | □ | □ | □ |
| For me, working is important. I want a job that I enjoy and that challenges me but that doesn't consume my entire life and leaves me time for other things. | □ | □ | □ | □ | □ |
| If it were financially possible, I could imagine working part-time and thus having more time for other things like family, friends, hobbies, etc. | □ | □ | □ | □ | □ |
| For me, having a job is not that important. I can well imagine dropping out of the workforce altogether and devoting myself to other things (i.e. family, friends, hobbies, etc). | □ | □ | □ | □ | □ |
| When I start a family and have young children, I would like to greatly reduce my professional activities. My family is more important to me. I can go back and work more later. | □ | □ | □ | □ | □ |

**How important to you is realization of the following life goals?**

I would like to…

|  | not important | low importance | somewhat important | important | very important |
| --- | --- | --- | --- | --- | --- |
| … continue educating myself | □ | □ | □ | □ | □ |
| … be around people a lot | □ | □ | □ | □ | □ |
| … live an exciting life | □ | □ | □ | □ | □ |
| … to advocate for others | □ | □ | □ | □ | □ |
| … have influence on others | □ | □ | □ | □ | □ |
| … have a deep relationship | □ | □ | □ | □ | □ |
| … broaden my horizons/mind | □ | □ | □ | □ | □ |
| … have a wide circle of acquaintances | □ | □ | □ | □ | □ |
| … experience adventures | □ | □ | □ | □ | □ |
| … act unselfishly | □ | □ | □ | □ | □ |
| … have high social status | □ | □ | □ | □ | □ |
| … give love and affection | □ | □ | □ | □ | □ |
| … constantly improve myself | □ | □ | □ | □ | □ |
| … do many things together with others | □ | □ | □ | □ | □ |
| … enjoy life to the fullest | □ | □ | □ | □ | □ |
| … do good | □ | □ | □ | □ | □ |
| … gain public recognition | □ | □ | □ | □ | □ |
| … have trusting relationships with others | □ | □ | □ | □ | □ |
| … develop my skills | □ | □ | □ | □ | □ |
| … have many social contacts | □ | □ | □ | □ | □ |
| … live an exciting life | □ | □ | □ | □ | □ |
| … help people in need | □ | □ | □ | □ | □ |
| … hold prestigious positions | □ | □ | □ | □ | □ |
| … receive affection and love | □ | □ | □ | □ | □ |

**For students aspiring to study human medicine:**

How do you see your future professional standing in 15 years?

□ General practitioner

□ Specialist in private practice

□ Hospitalist

□ Medical research

□ Management/ consultancy

□ Medical informatics/ medical technology

□ Insurance medicine/ public health

□ Undecided

□ Other, please specify: __________________ [free text response]

What fascinates my about studying human medicine is: _____________________ [free text response]

The official weekly work time for residents is currently 50 hours (including night duties and weekend duties). What is your opinion on this?

□ No opinion on this

□ I don’t see any problem with the weekly work time; this is part of the job

□ I find the weekly work time too much. I would find the following weekly work time appropriate (please specify number): ___
